# Supplementary material for: Seminoma and Embryonal Carcinoma Footprints Identified by Analysis of Integrated Genome-Wide Epigenetic and Expression Profiles of Germ Cell Cancer Cell Lines
Source: PLoS One. 2014 Jun 2;9(6):e98330. doi: 10.1371/journal.pone.0098330 (PMC4041891; doi:10.1371/journal.pone.0098330)
Supplement: File S1 — ZIP file containing DMRforPairs output for significant regions. Please start from the html files. (ZIP) [file pone.0098330.s008.zip › figures/28382.pdf]

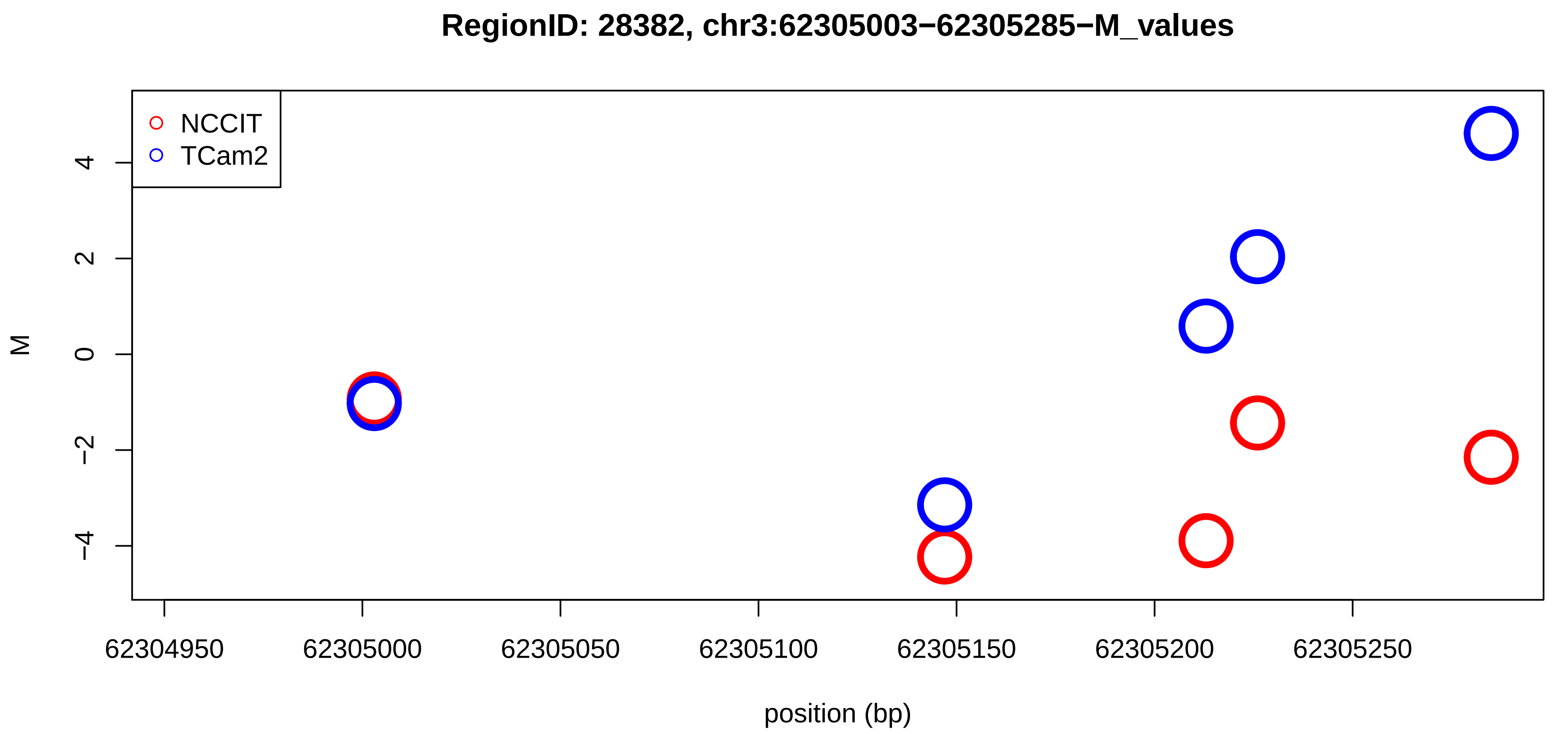
RegionID: 28382, chr3:62305003−62305285−M_values
NCCIT
TCam2
M
4
2
0
−2
−4
62304950
62305000
62305050
62305100
62305150
62305200
62305250
position (bp)

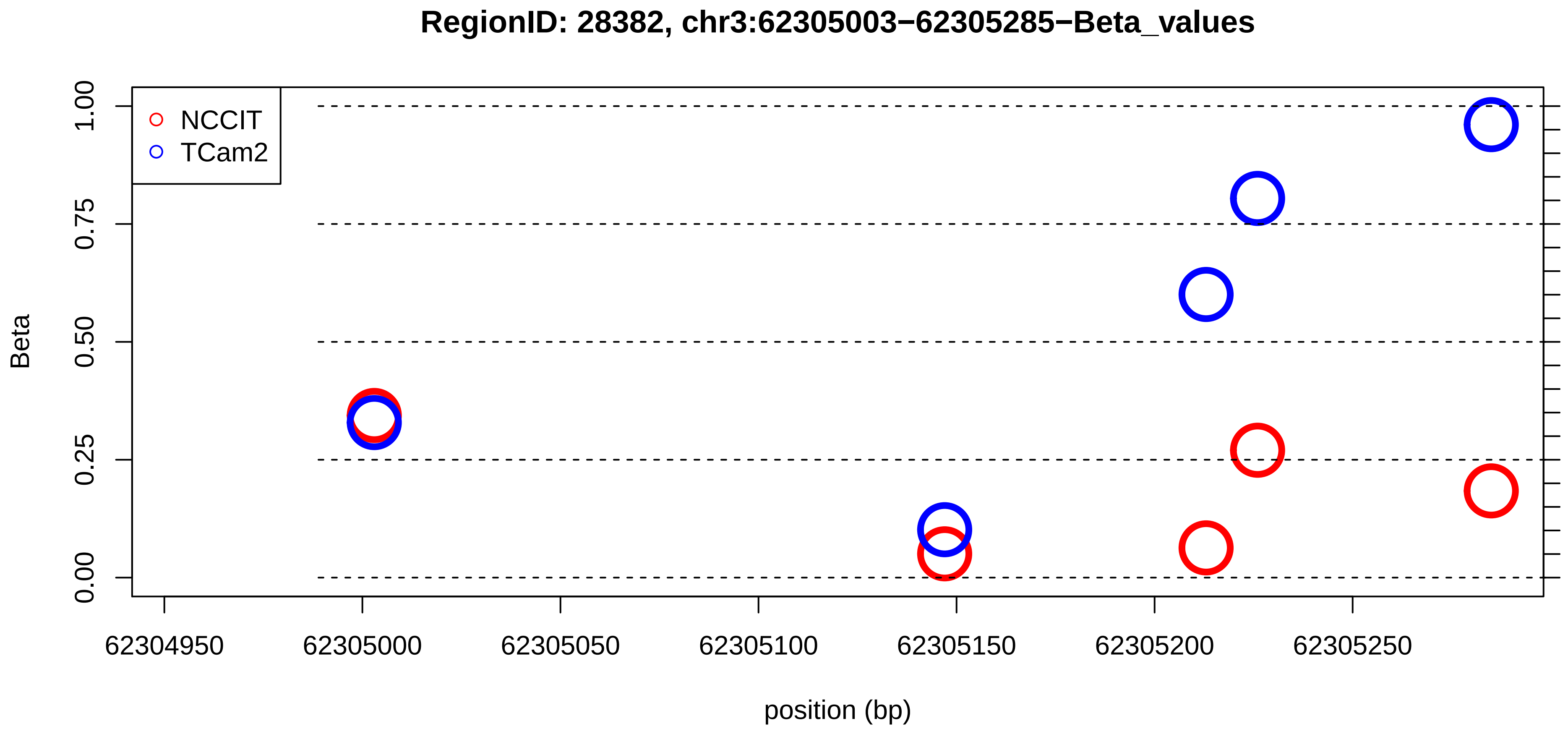
RegionID: 28382, chr3:62305003−62305285−Beta_values
NCCIT
TCam2
Beta
1.00
0.75
0.50
0.25
0.00
62304950
62305000
62305050
62305100
62305150
62305200
62305250
position (bp)
